# A clinical audit of the electronic data capture of dementia in ambulance service patient records

**DOI:** 10.29045/14784726.2018.03.2.4.10

**Published:** 2018-03-01

**Authors:** Helen Pocock, Patryk Jadzinski, Chloe Taylor-Jones, Phil King, Ed England, Carole Fogg

**Affiliations:** South Central Ambulance Service NHS Foundation Trust; South Central Ambulance Service NHS Foundation Trust; University of Portsmouth; South Central Ambulance Service NHS Foundation Trust; South Central Ambulance Service NHS Foundation Trust; South Central Ambulance Service NHS Foundation Trust; University of Portsmouth; Portsmouth Hospitals NHS Trust

**Keywords:** clinical audit, dementia, emergency medical services

## Abstract

**Background:**

Dementia is a common diagnosis in older people. It is important to identify and record dementia on emergency call-outs, as it impacts on subsequent care decisions. Ambulance services are changing from paper to electronic patient records, but there are limited data on how frequently and in which sections of the electronic patient record dementia is being recorded.

**Aims:**

To audit the proportion of ambulance electronic patient records where dementia is recorded for patients aged (i) 65 and above and (ii) 75 and above, and to describe the sections in the electronic patient record in which dementia is recorded, as there is currently no standardised button or field available.

**Results:**

A total of 314,786 electronic patient records were included in the audit, over a one-year period. The proportion of attended calls with ‘dementia’ recorded in the electronic patient record in patients aged 65+ was 13.5%, increasing to 16.5% in patients aged 75+, which is similar to that recorded in previous literature. For patients aged 75+ conveyed to hospital, 15.2% had ‘dementia’ recorded in the electronic patient record, which may indicate under-recording. Recording of dementia between Clinical Commissioning Groups varied between 11.0% and 15.3%. Dementia was recorded in 16 different free-text fields, and 38.4% of records had dementia recorded in more than one field.

**Conclusion:**

This audit demonstrates high variability in both the frequency of recording dementia and also the location in the electronic patient record. To ensure consistent recording and ease of retrieval to inform patient care and handover, we propose that the electronic patient record should be modified to reflect paramedics’ needs, and those of the healthcare staff who receive and act on the report. Enhanced training for paramedics in the importance and method of recording dementia is required. Future data will enable accurate monitoring of trends in conveyance, and inform justifications for alternative services and novel referral pathways.

## Background

The number of people living with dementia is increasing as the UK population ages (Alzheimer’s Society, 2017). An estimated one in 23 people aged 65 years and above (65+) have dementia (NHS Digital, 2017).

People with dementia with ambulatory-care sensitive conditions and acute episodes of illness are more likely to be admitted to hospital than people without dementia (Phelan, Borson, Grothaus, Balch, & Larson, 2012). In 2016, 207,797 patients aged 65+ with a diagnosis of dementia were admitted to hospital in an emergency in the UK (NHS Digital, 2017). A recent analysis of 19,269 patients aged 75 years and above (75+) screened for dementia at hospital admission revealed that 19.8% of patients had a dementia diagnosis, and an additional 11.6% of patients had cognitive impairment, which may include undiagnosed or unreported dementia (Fogg, Meredith, Bridges, Gould, & Griffiths, 2017). Around 5.5% of patients initially treated on scene by the emergency medical services (EMS) subsequently re-contact 999 within 24 hours (HSCIC, 2015), suggesting an initial incomplete assessment of needs. Knowledge of the presence of dementia is important so that appropriate care can be provided such as dementia-friendly communication and support with food and fluid intake as well as referral to supportive tertiary services.

The increase in the number of people living with dementia also impacts on emergency, out-of-hospital care. This is reflected in the addition of a section on ‘Dementia, Alzheimer’s, Parkinson’s disease and palliative care’ in the most recent edition of the College of Paramedics’ Paramedic Curriculum Guidance (Harris, 2013). It is essential to assess the extent to which care needs of this patient group are being met in pre-hospital care. Data from a large acute hospital within the South Central region suggest that people with identified cognitive impairment may have similar enhanced care needs to those with a formal dementia diagnosis, with an increased risk of mortality during hospitalisation among patients aged 75+ with dementia or cognitive impairment when compared to those without (10.8% and 11.8% v. 6.6%) (Fogg et al., 2017). However, the role of the EMS in caring for older people with dementia is not clearly understood in the literature. An integrative review identified key themes as providing emergency transport, assessment and treatment, and as a ‘last resort’ or safety net for providing care (Buswell, Lumbard, Prothero et al., 2016).

### Outline

Little is currently known about the ability of the EMS to accurately record dementia prevalence across their service users. An analysis of 358 ambulance paper patient care records (PCRs) of patients aged 75+ found that ‘dementia’ was recorded in 14.5% (95% CI 10.9%—18.2%) of PCRs, with a lower than expected proportion of dementia recorded for care home residents (Buswell, Lumbard, Fleming et al., 2016). The lack of systematic recording of dementia due to no specific ‘tick box’ on the form means it is not possible to say whether the latter was an accurate reflection of the study population or whether dementia was under-recognised or under-reported. Dementia was recorded across a range of data fields including previous medical history, social or family history and treatment advice or notes.

To enable more systematised and individualised recording of patient data, ambulance services are moving towards electronic recording. This provides opportunities for improvement in record keeping, audit and quality of handover or referral information. However, capture and storage of electronic patient information within ambulance services present challenges, and the current usage and potential for electronic patient records (EPRs) to facilitate hospital avoidance are currently under study (Porter et al., 2016). There is currently no published large-scale evaluation of how frequently dementia is being recorded in the EPR within an ambulance service region, how this varies within a region and where it is commonly recorded. This information is important to ascertain the completeness and location of dementia recording to optimise the design of electronic recording systems and inform staff training on their use.

### Aim

This audit aims to report the proportion of ambulance EPRs where dementia was recorded for patients aged 65+ — that is, when the prevalence of dementia becomes significant. It also compares the proportion of ambulance records with dementia in patients aged 75+ with published literature for attended ambulance calls, and those conveyed to hospital with estimates of dementia prevalence in hospitalised patients. The fields in the EPR where dementia is recorded will also be described.

### Objectives of audit

- To describe the proportion of EPRs with ‘dementia’ recorded in EMS attendances to people aged 65+.
- To ascertain whether the proportion of EPRs with a record of ‘dementia’ in EMS attendances to people aged 75+ is similar to that found in a paper records audit (Buswell, Lumbard, Fleming et al., 2016).
- To ascertain whether the proportion of EPRs with ‘dementia’ recorded in EMS attendances to people aged 75+ who are conveyed to hospital is similar to the proportion of patients with dementia recorded via a systematic dementia screening system in a large district general hospital within the operational area (Fogg et al., 2017).
- To describe the frequency and distribution of the fields of the EPR in which ‘dementia’ is being recorded.

## Methods

### Standards, guidelines and evidence base

There are currently no pre-hospital dementia-screening guidelines or standards.

### Sample: patient population and setting

Although published statistics regarding dementia include patients aged 65+, dementia increases in prevalence with age. The population of interest was patients aged 65+ with an emergency call attended by the EMS between 1 April 2016 and 31 March 2017 inclusive with an EPR completed. The subset of patients aged 75+ within this population was analysed to enable comparison with published literature. We only considered calls attended within the geographical boundaries of the South Central Ambulance Service (SCAS) NHS Foundation Trust (Figure 1).

**Figure:**
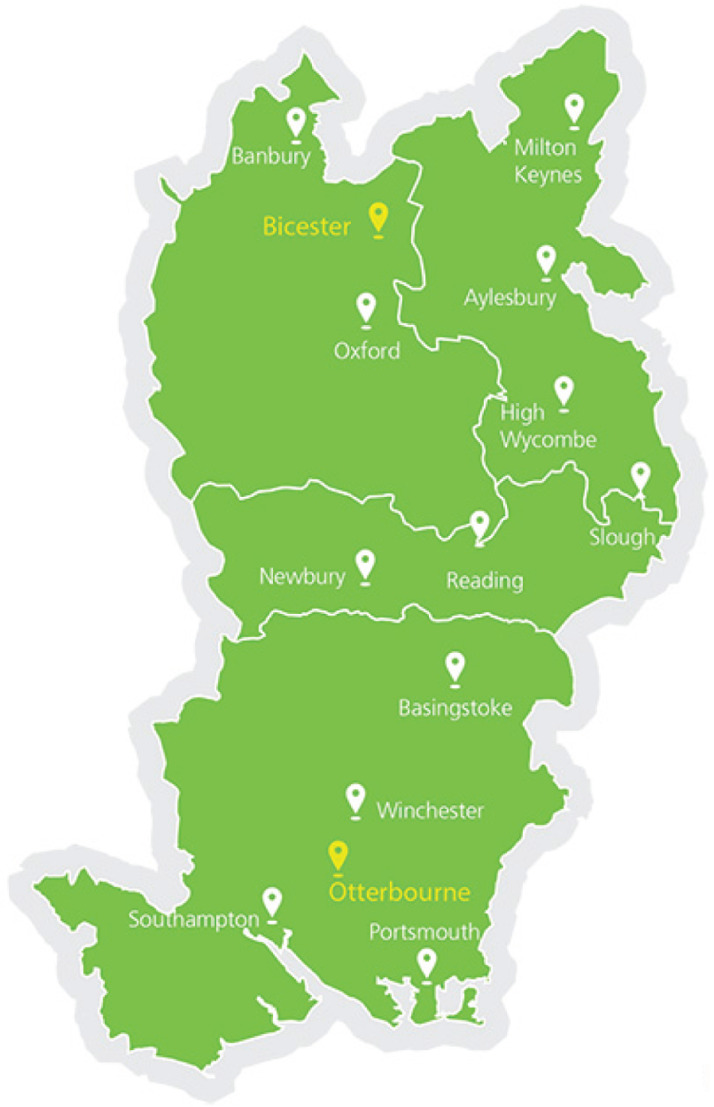
Figure 1. Geographical area covered by South Central Ambulance Service (SCAS).

### Data source

Data were collected during routine care via an EPR on the MobiMed Smart electronic tablet (Ortivus, Sweden). Each patient attended by the emergency crew or a solo responder had an EPR completed each time they were attended by the EMS. The EPR is designed to collect patient clinical and social history, incident details and clinical information, for example vital signs and clinical diagnostics (e.g. electrocardiogram (ECG) data). Data are entered into the EPR via a touchscreen on a tablet at the scene, through menus and interactive, self-expanding boxes as well as sections of free text, where additional detail about the examination is deemed necessary. Each patient record collects the same information that the crew would have had an opportunity to document on a paper record form, thus no additional paper records are required or used by the ambulance teams.

### Audit type

The audit was reviewed and approved by the SCAS Clinical Review Group in December 2016.

## Methods

### Data management and extraction

The tablet uses the mobile network and wireless internet connection to transfer data to a data warehouse on a server located in the UK (managed by Hytek). A copy of the data is downloaded daily to the Ambulance Trust Business Intelligence Team, and stored within local databases (Dochaven and Total Clinical Management Patient Report Form) on a secure server.

Microsoft SQL Server Management Studio was used to extract data from the SCAS data warehouse. Free-text fields to be searched were identified by SCAS staff performing a mock data entry exercise and attaining consensus on which fields could reasonably have ‘dementia’ recorded, within the context of the question. Queries were written to identify when the term ‘dementia’ was present within free-text fields, to ensure anonymity was achieved by hiding free text that could contain identifiable information.

Additional contextual data from several data sources, such the Clinical Commissioning Group (CCG) area, are accessible via Qlikview, a ‘presentation layer’ application. The EPR dataset was uploaded into a standalone application within which incident numbers were matched with the Computer Aided Dispatch (CAD) information, and transferred to a Microsoft Excel spreadsheet.

### Data analysis

Descriptive, aggregate tables were formed using Microsoft Excel (2010). The total number of emergency attendances where an EPR was used, as well as that for patients (i) aged 65+ and (ii) aged 75+, and those with a record of dementia, were summarised by CCG within the SCAS area. The number and proportion of patients aged 65+ and 75+ with a dementia record who were conveyed and not conveyed were calculated. The frequency of the word ‘dementia’ by data entry field on the EPR was retrieved, and the proportion of total patient records with ‘dementia’ in that field was calculated. Confidence intervals (CIs) and odds ratios were calculated using Stata version 13.1, College Station, Texas (2013).

### Caveats

The total number of clinical records produced in SCAS within this period was 459,086. To enable meaningful between-areas comparisons, only areas where more than 70% of clinical records were electronic were included (Figure 2). Only those emergency calls attended and recorded by core SCAS staff were included. The four CCGs removed from this analysis are more dependent on private providers, who use paper records. In these areas, paper records are kept both by conveying crews who are first on the scene and by ambulance service first responders, who hand over care to a private crew. The number of records excluded from this audit was 144,300. However, the large dataset from the remaining 13 CCGs is likely to be representative of the remaining areas.

**Figure:**
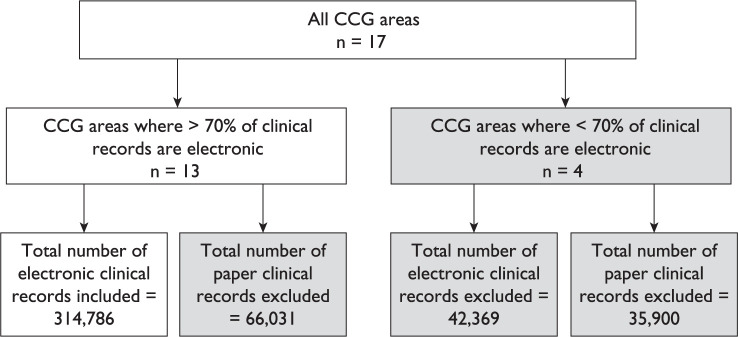
Figure 2. Audit profile.

## Results

Of the 17 CCGs in the geographical area, 13 had more than 70% of attendances with an EPR record completed. Of the attendances in these 13 CCGs, 47.1% (n = 148,255) were to patients aged 65+, and 35.4% (n = 111,548) to patients aged 75+ (Table 1). The proportion of attendances for patients aged 65+ compared to total attendances varied across CCGs, from 36.7% in Southampton CCG to 55.4% in West Hampshire. The proportion of attendances in West Hampshire and Oxfordshire, the CCGs with the largest elderly population, was 39.8% (Office for National Statistics, 2016).

**Table 1.** Occurrence of emergency ambulance attendances to people aged 65+ and 75+ and frequency of ‘dementia’ record in free text, by Clinical Commissioning Group Area.

CCG	Total attendances within CCG with EPR (n)	Attendances for patients aged 65+			65+ with a dementia record		
		(n)	Proportion per CCG (%)	Proportion of all CCGs (%)	(n)	Proportion per CCG (%)	Proportion of all CCGs (%)
**Patients aged 65+**							
Aylesbury Vale CCG	14,903	7095	47.6	4.8	915	12.9	4.6
Bracknell and Ascot CCG	10,538	4430	42.0	3.0	486	11.0	2.4
Fareham and Gosport CCG	19,923	10,486	52.6	7.1	1457	13.9	7.3
Milton Keynes CCG	25,167	9850	39.1	6.6	1348	13.7	6.7
Newbury and District CCG	10,273	4819	46.9	3.3	687	14.3	3.4
North & West Reading CCG	7563	3823	50.5	2.6	500	13.1	2.5
North Hampshire CCG	18,451	8561	46.4	5.8	1087	12.7	5.4
Oxfordshire CCG	62,721	28,432	45.3	19.2	3498	12.3	17.5
Portsmouth CCG	25,924	10,837	41.8	7.3	1660	15.3	8.3
South Eastern Hampshire CCG	23,369	12,877	55.1	8.7	1725	13.4	8.6
Southampton CCG	30,363	11,152	36.7	7.5	1529	13.7	7.6
West Hampshire CCG	54,984	30,481	55.4	20.6	4295	14.1	21.5
Wokingham CCG	10,607	5412	51.0	3.7	827	15.3	4.1
**Total/overall (%)**	**314,786**	**148,255**	**47.1**	**100.0**	**20,014**	**13.5**	**100.0**
**Patients aged 75+**							
Aylesbury Vale CCG	14,903	5256	35.3	4.7	837	15.9	4.5
Bracknell and Ascot CCG	10,538	3269	31.0	2.9	440	13.5	2.4
Fareham and Gosport CCG	19,923	7975	40.0	7.1	1325	15.6	7.2
Milton Keynes CCG	25,167	7002	27.8	6.3	1206	16.6	6.6
Newbury and District CCG	10,273	3586	34.9	3.2	628	17.2	3.4
North & West Reading CCG	7563	2901	38.4	2.6	456	17.5	2.5
North Hampshire CCG	18,451	6325	34.3	5.7	983	15.7	5.3
Oxfordshire CCG	62,721	21,540	34.3	19.3	3256	15.5	17.7
Portsmouth CCG	25,924	7973	30.8	7.1	1489	15.1	8.1
South Eastern Hampshire CCG	23,369	9833	42.1	8.8	1573	18.7	8.5
Southampton CCG	30,363	8050	26.5	7.2	1389	15.1	7.5
West Hampshire CCG	54,984	23,574	42.9	21.1	4045	16.0	22.0
Wokingham CCG	10,607	4264	40.2	3.8	777	19.2	4.2
**Total/overall (%)**	**314,786**	**111,548**	**35.4**	**100.0**	**18,404**	**16.5**	**100.0**

### Frequency of recording of dementia and rates of conveyance

Dementia was recorded in 13.5% (95% CI 13.3—13.7) (n = 20,014) of attendances in patients aged 65+, and 16.5% (95% CI 16.3—16.7) (n = 18,404) of those aged 75+ (Table 1). Patients aged 75+ contributed 92.0% of the total records with dementia. The proportion of 65+ with a dementia record varied between CCGs, from 11.0% in Bracknell and Ascot CCG to 15.3% in Portsmouth CCG and Wokingham CCG.

Patients with a record of ‘dementia’ in the EPR had lower conveyance rates than those with no dementia record (Table 2). There was an association between a recording of dementia and a lower odds of being conveyed to hospital for patients aged 65+ (odds ratio (OR) 0.73, 95% CI 0.71—0.75) and also for patients aged 75+ (OR 0.77, 95% CI 0.75—0.80).

**Table 2.** Frequency of ‘dementia’ record in patients conveyed to hospital, by Clinical Commissioning Group Area, for patients aged 65+.

CCG	Total attendances 65+ or 75+(n)	Total conveyed		Total records with dementia (n)	Patients with dementia conveyed			Patients with no record of dementia (n)	Patients with no record of dementia conveyed	
		(n)	(%)		(n)	(%)	Proportion of total conveyed (%)		(n)	(%)
**Patients aged 65+**										
Aylesbury Vale CCG	7095	4913	69.2	915	587	64.2	11.9	6180	4326	70.0
Bracknell and Ascot CCG	4430	3013	68.0	486	337	69.3	11.2	3944	2676	67.8
Fareham and Gosport CCG	10,486	6710	64.0	1457	843	57.9	12.6	9029	5867	65.0
Milton Keynes CCG	9850	6699	68.0	1348	900	66.8	13.4	8502	5799	68.2
Newbury and District CCG	4819	2972	61.7	687	369	53.7	12.4	4132	2603	63.0
North & West Reading CCG	3823	2469	64.6	500	291	58.2	11.8	3323	2178	65.5
North Hampshire CCG	8561	5332	62.3	1087	556	51.1	10.4	7474	4776	63.9
Oxfordshire CCG	28,432	18,746	65.9	3498	2118	60.5	11.3	24,934	16,628	66.7
Portsmouth CCG	10,837	6817	62.9	1660	924	55.7	13.6	9177	5893	64.2
South Eastern Hampshire CCG	12,877	8157	63.3	1725	985	57.1	12.1	11,152	7172	64.3
Southampton CCG	11,152	7726	69.3	1529	1003	65.6	13.0	9623	6723	69.9
West Hampshire CCG	30,481	19,532	64.1	4295	2379	55.4	12.2	26,186	17,153	65.5
Wokingham CCG	5412	3549	65.6	827	487	58.9	13.7	4585	3062	66.8
**Total/overall (%)**	**148,255**	**96,635**	**65.2**	**20,014**	**11,779**	**58.9**	**12.2**	**128,241**	**84,856**	**66.2**
**Patients aged 75+**										
Aylesbury Vale CCG	5256	3600	68.5	837	542	64.8	15.1	4419	3058	69.2
Bracknell and Ascot CCG	3269	2180	66.7	440	299	68.0	13.7	2829	1881	66.5
Fareham and Gosport CCG	7975	4973	62.4	1325	759	57.3	15.3	6650	4214	63.4
Milton Keynes CCG	7002	4687	66.9	1206	800	66.3	17.1	5796	3887	67.1
Newbury and District CCG	3586	2156	60.1	628	332	52.9	15.4	2958	1824	61.7
North & West Reading CCG	2901	1798	62.0	456	266	58.3	14.8	2445	1532	62.7
North Hampshire CCG	6325	3810	60.2	983	502	51.1	13.2	5342	3308	61.9
Oxfordshire CCG	21,540	14,007	65.0	3256	1961	60.2	14.0	18,284	12,046	65.9
Portsmouth CCG	7973	4902	61.5	1489	836	56.1	17.1	6484	4066	62.7
South Eastern Hampshire CCG	9833	6122	62.3	1573	889	56.5	14.5	8260	5233	63.4
Southampton CCG	8050	5443	67.6	1389	912	65.7	16.8	6661	4531	68.0
West Hampshire CCG	23,574	14,674	62.2	4045	2238	55.3	15.3	19,529	12,436	63.7
Wokingham CCG	4264	2727	64.0	777	462	59.5	16.9	3487	2265	65.0
**Total/overall (%)**	**111,548**	**71,079**	**63.7**	**18,404**	**10,798**	**58.7**	**15.2**	**93,144**	**60,281**	**64.7**

Conveyance rates varied between CCGs in a similar pattern whether the patient was noted to have dementia or not. There was far greater variability where the patient had dementia recorded.

Patients with a dementia record comprised 12.2% (95% CI 12.0—12.4) of the total conveyances of patients aged 65+, rising to 15.2% (95% CI 14.9—15.5) for patients aged 75+.

### Location of recording of dementia on the EPR

The distribution of the EPR data entry fields where dementia was recorded is displayed in Table 3. There was a total of 29,984 records of the word ‘dementia’ within 20,014 patient EPRs, thus 38.4% of EPRs had ‘dementia’ recorded in more than one data collection field. The most common field used to record dementia was ‘other known medical history’, with 45.6% of all EPRs with a dementia record having ‘dementia’ entered in this field. However, 25.5% (n = 7687) of EPRs had dementia recorded under the presenting condition free-text box, with a further 21.0% recorded in the ‘other known neurological medical history’. For 305 (0.21%) EPRs, the input record for dementia contained a ‘?’ (indicating a diagnosis was not confirmed) in at least one field, for example ‘?dementia’ or ‘dementia?’.

**Table 3.** Distribution of ‘dementia’ text according to data entry field in the EPR.

EPR section	Sub-section	Free-text data collection field	Frequency of ‘dementia’ recorded (n = 29,984)	65+ records with ‘dementia’ present in EPR with ‘dementia’ in this field (N = 20,014)
Incident	Patient	Social history notes	413	2.1%
	Presenting condition	Presenting condition/chief complaint (free text following main ‘choice’ field)	5098	25.5%
AMPLE*	Past medical history	Other known medical history	9128	45.6%
		Other known neurological medical history	4207	21.0%
Examination	Mental health	Mental health notes	3794	19.0%
		Mental health diagnosis — further information	61	0.3%
		Mental health diagnosis — notes	22	0.1%
	General	Brief description of injury or illness	3123	15.6%
		Signs and symptoms	1160	5.8%
		What are the clinician’s concerns? (relating to falls risk assessment concerns)	325	1.6%
		Previous medical history including falls (any patterns?) (relating to falls risk assessment past medical history)	1091	5.5%
		Patients confirmed diagnosed conditions (relating to falls risk assessment confirmed/diagnosed)	1088	5.4%
Final disposition	Impression and plan	Further information regarding diagnosis (‘impression differential diagnosis’)	101	0.5%
		Impression	251	1.3%
		Impression working diagnosis	72	0.4%
	Non-conveyance	Diagnosis notes (relating to ‘SeeTreatFreetext’)	50	0.2%

*AMPLE = Allergies, Medications, Past medical history, Last oral intake, Events leading to.

## Discussion

The proportion of records with ‘dementia’ recorded in the EPR rose from 13.5% in patients aged 65+ to 16.5% in patients aged 75+, reflecting the increasing prevalence and diagnosis of dementia with age. In patients aged 75+, the prevalence of 16.5% of records noting dementia in the EPR was higher than the 14.5% found on paper records (Buswell, Lumbard, Fleming et al., 2016). This may reflect greater precision of the estimate due to larger sample size, increased emphasis on earlier diagnosis or greater awareness among ambulance crews following local training activities. Alternatively, the greater number of data fields on EPR prompts staff to include more information, enabled by multiple forms on the EPR rather than the traditional single A5 sheet of paper.

The proportion of conveyed patients aged 75+ with a dementia record (15.2%) is slightly lower than that of the acute hospital dementia screening programme of 19% (Fogg et al., 2017). The higher prevalence in hospital could reflect the systematic nature of the screening process, and that dementia diagnoses may not be disclosed during pre-hospital assessment. Additionally, hospital screening occurs after admission, therefore not including patients who attend the emergency department and are subsequently discharged. Introduction of systematic screening in the pre-hospital environment may benefit patients who are not conveyed or not admitted to hospital, as a missed dementia diagnosis would mean these patients may miss out on additional services to meet their enhanced care needs. In this audit, less than 1% of records reflected uncertainty about the dementia diagnosis. With additional training and the introduction of screening, more patients may be identified and staff confidence in recording increased.

Variation in the proportion of patients identified with dementia by CCG may be influenced by several factors. At the time of the audit, training on dementia across the area was ongoing, therefore crews may be more alert to detecting and recording dementia, for example by finding information in available care notes. There may also be differences in the way that teams were trained to complete the EPR, and variability in dementia diagnosis rates across the country related to training and availability of services in the primary care trusts in the CCGs in which the crews are operating. Areas with higher densities of older persons may have different provision for out-of-hours care or higher densities of nursing/residential homes, accounting for variation in conveyance rates. This is supported by a previous finding that only 43% of calls from care homes resulted in an unscheduled hospital admission (Amador et al., 2014). Additionally, local adaptations to services, for example the Acute Frailty Intervention Team (AFIT) based in West Hampshire, could impact on the quality of assessment of patients in the community, and the emphasis on reporting of dementia.

Dementia commonly contributes to frailty in older people (BGS, 2014). The SCAS EPR offers the function of recording a frailty score based on the Rockwood scale. A recent audit of patient frailty scores revealed similar levels of prevalence between those patients conveyed to hospital and those discharged at the scene (Cavalier, 2017). Patients rated ‘very fit’, ‘well’ or ‘managing well’ were more likely to be admitted to hospital than discharged at the scene (32% v. 26%), whereas patients rated ‘moderately frail’ to ‘terminally ill’ were less likely to be admitted to hospital (41% v. 47%). These results are congruent with our observations of dementia conveyance, and highlight the importance of both groups of patients having access to appropriate care in the community to avoid repeat ambulance call-outs and re-attendances. Our results contrast with Phelan et al. (2012) whose study population was in the US and may simply reflect the differences in healthcare provision.

The finding that dementia was recorded in more than one field in 38.4% of cases suggests that there is considerable inefficiency in the completion of the EPR. If staff are unfamiliar with the appearance of the printed clinical record they may duplicate information to ensure it will not be missed. Alternatively, they may believe that recording information in every possible field will facilitate audit. If dementia is being recorded in multiple fields it is likely that other information is also being duplicated, thus increasing the inefficiency of EPR completion.

A relatively low recording rate of dementia could be found in the falls assessment section of the EPR. This may be due to the fact that clinicians had already recorded it elsewhere or that they failed to identify dementia as a risk factor for falls. As well as closed response options regarding specific risk factors (including confirmed diagnoses), there are free-text fields within this section. Given that sensory or visual disturbance is commonly associated with dementia, this may highlight a staff education need. The most common field where dementia was entered was ‘other known medical history’. This category is generally used to record less significant medical history, which may lead to the dementia being overlooked. In around a quarter of cases where dementia was recorded, it was located in the ‘presenting condition’ field, suggesting a greater significance for the condition, possibly due to increased severity of dementia or greater impact of dementia on the current situation. Overall, the analysis of the EPR fields suggests a lack of clarity about when and where dementia should be recorded.

### Recommendations/learning points

- The EPR should be modified to reflect para medics’ needs, and those of the healthcare staff who receive and act on the report.
- Enhanced training for paramedics in the importance and method of recording dementia is required.

### Limitations

There is the potential for misclassification as to the recording of ‘dementia’ in the EPR, as it may apply to the patient’s spouse or in the case that the patient is a carer for someone with dementia. However, we consider this may happen in a limited number of cases, as staff are trained to complete the EPR from the patient perspective. Additionally, the time taken and level of detail in the EPR may differ according to whether the patient is taken to hospital or not and the urgency of the conveyance, with more detailed notes written for patients staying at home, and an increasing likelihood of the notes having ‘dementia’ recorded, which may also lead to differential misclassification and may explain the higher proportion of patients with EPRs containing ‘dementia’ not being conveyed. This audit does not include dementia records in patients aged less than 65 years, as dementia is less common in this age group and also less likely to be diagnosed.

The analysis included only those sections of the EPR the team considered likely to include a record of ‘dementia’, rather than the whole EPR. However, the authors feel that this is likely to have minimal impact on non-detection of ‘dementia’.

## Author contributions

All authors were involved in the design of the audit. PK extracted the data and PK and CF performed data analysis. All authors were involved in interpretation of data, and in the writing of the manuscript. All authors have approved the final manuscript.

## Conflict of interest

None declared.

## Funding

South Central Ambulance Service NHS Foundation Trust; University of Portsmouth; Portsmouth Hospitals NHS Trust.
